# Nanomechanical humidity detection through porous alumina cantilevers

**DOI:** 10.3762/bjnano.6.137

**Published:** 2015-06-16

**Authors:** Olga Boytsova, Alexey Klimenko, Vasiliy Lebedev, Alexey Lukashin, Andrey Eliseev

**Affiliations:** 1 Lomonosov Moscow State University, 119992, Moscow, Russia; 2 Kurnakov Institute of General and Inorganic Chemistry of The Russian Academy of Science, 119991, Moscow, Russia

**Keywords:** anodic aluminium oxide, atomic force microscopy (AFM), cantilever arrays, humidity, mechanical sensor, porous alumina

## Abstract

We present here the behavior of the resonance frequency of porous anodic alumina cantilever arrays during water vapor adsorption and emphasize their possible use in the micromechanical sensing of humidity levels at least in the range of 10–22%. The sensitivity of porous anodic aluminium oxide cantilevers (Δ*f*/Δ*m*) and the humidity sensitivity equal about 56 Hz/pg and about 100 Hz/%, respectively. The approach presented here for the design of anodic alumina cantilever arrays by the combination of anodic oxidation and photolithography enables easy control over porosity, surface area, geometric and mechanical characteristics of the cantilever arrays for micromechanical sensing.

## Introduction

The last two decades have seen a surge in resonant micro- and nanomechanical engineering raised by the creation of high-performance and cost-effective detection systems. Highly elastic silicon and, in contrast, soft polymer cantilevers were implemented to chemical sensors with resonance shift assay [1–3]. To date, the determination of trace amounts of adsorbed biological, chemical and other multiplex substances through micromechanical sensing still holds a tremendous potential [4–8]. To improve the shifts of the resonant frequency one needs to enlarge the active surface area of the sensor while preserving its mechanical stiffness. This necessitates the use of the porous systems with “open through porosity”. One promising porous system exhibiting all the necessary characteristics is anodic aluminium oxide (AAO). Its huge surface area offers an enormous number of binding sites.

The adsorption of chemicals on the surface of the cantilever manifests itself by the shift of the resonance frequency in dynamic vibration mode. The loading mass can be calculated from the change of resonance frequencies after (ƒ_1_) and before (ƒ_0_) adsorption as follows [9–10]:

[1]
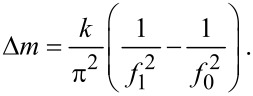


Here, the spring constant *k* is given by *k* = (*E*·*w*·*t*^3^)/(4·*l*^3^), where *w*, *t* and *l* are the width, thickness and length of the cantilever and *E* is the Young’s modulus of the cantilever [11]. According to Equation 1, the more molecules are adsorbed on the surface of a cantilever, the larger is the shift of the resonance frequency. Therefore AAO cantilevers hold a great promise for the development of micromechanical sensor arrays.

In the present work we concentrated on the elaboration of experimental methods for the fabrication of anodic alumina cantilever arrays and their testing for adsorption of water vapors.

## Results and Discussion

**Figure 1:**
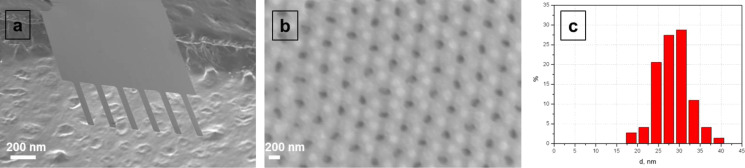
SEM images of a six-cantilevers array (2 μm thickness) obtained through chemical photolithography: (a) general view, (b) porous structure of AAO cantilever with 30 nm average pore diameter and 105 nm average interpore distances and (c) pore size distribution.

The proposed procedure for the fabrication of cantilever arrays combines anodic oxidation of aluminium foil with a conventional photolithography process (Figure 1).

Using the lithography approach for AAO pattern formation mostly becomes possible due to selective and anisotropic etching of anodic alumina films on aluminium in basic solutions. On the other hand anodic oxidation itself enables easy control over channel diameter and interpore distance of the porous film over a wide range (pore diameter from 15 to 200 nm; interpore distance from 50 to 500 nm, film thicknesses up to 200 micrometers) [3]. Moreover the thickness of AAO films can be regulated precisely by using conventional electrochemistry methods by controlling the total electric quantity and assuming a current efficiency of 90% [12]. Thus, the proposed approach enables easy control over porosity, surface area, and geometric characteristics of cantilever arrays which provide wide opportunities for the design of micromechanical sensors with specific mechanical response.

**Figure 2:**
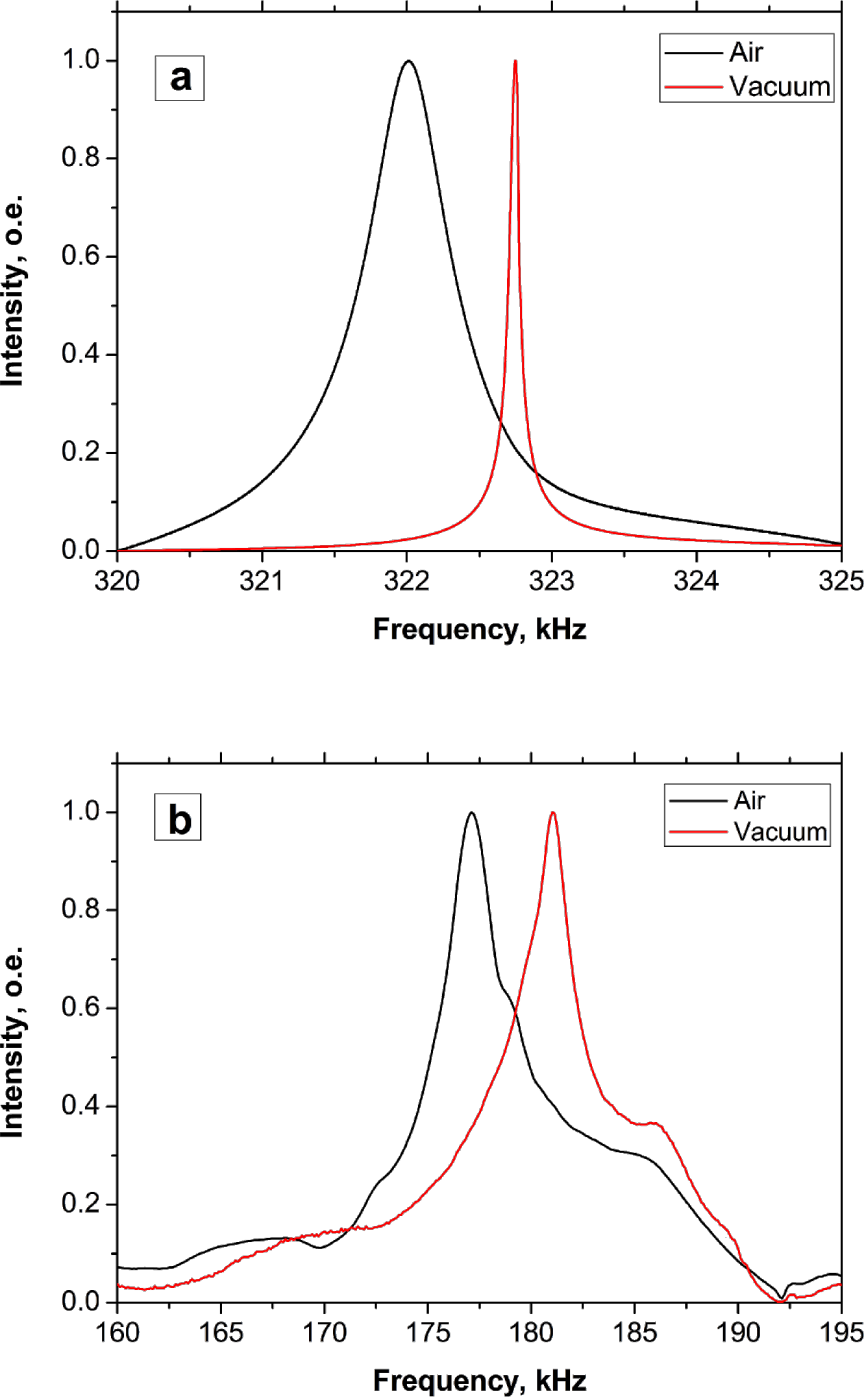
a) Frequency responses of Si rectangular cantilever (2 μm thick, 100 μm long and 30 μm wide) in the air (black line) and in vacuum (red solid line). The frequency shift is 780 Hz. b) Frequency responses of AAO cantilever (2 μm thick, 800 μm long and 100 μm wide) in the air (black line) and in vacuum (red solid line). The shift here is 3900 Hz.

To predict the behavior of the cantilever in the gas/liquid phase we investigated alterations of the amplitude–frequency characteristics when changing pressure and humidity. The frequency response of elastic beam is strongly dependent on the fluid it is vibrating in [2]. In vacuum, when the vapors are absent, the measurement of the resonance frequency is routinely made compared to measurements in viscous media. At first we emphasized the damping effect on cantilever vibration for porous AAO and standard Si cantilevers explored in the real system (air) and in model (vacuum). The amplitude–frequency profiles of rectangular cantilevers made of Si and porous anodic aluminium oxide are provided in Figure 2. Their characteristics are summarized in Table 1. The *Q* factor for both types of beams significantly increases after change air media to vacuum. Natural shifts to high-frequency region are observed for the porous cantilever as well for the Si cantilever.

**Table 1:** Shifts of resonance frequency and quality factors *Q* for Si and AAO cantilevers under different pressure (vacuum and air, humidity 22%).

cantilever material	Si		AAO	

condition	air	vacuum (10^−5^ mbar)	air	vacuum (10^−5^ mbar)
*Q* factor	350	3230	39	52
resonant frequency *f*, kHz	322.01	322.79	177.12	181.02

**Figure 3:**
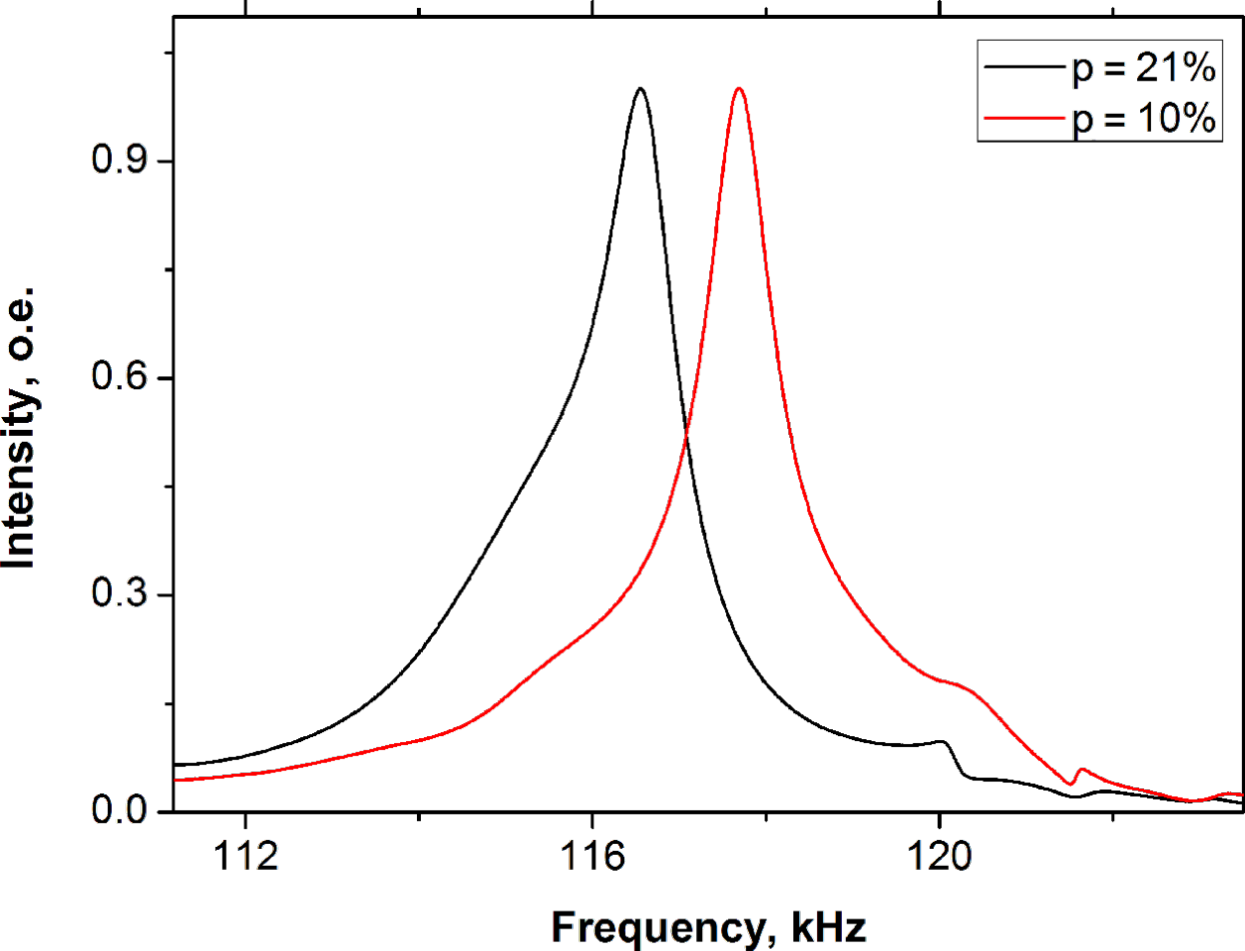
Frequency responses of AAO rectangular cantilever (2 μm thick, 800 μm long and 100 μm wide) at relative humidities of 10 and 21% (1120 Hz shift).

For comparison we also studied the effect of the water content on the vibration of the cantilever from the AAO membrane obtained at 40 V in 0.3 M oxalic acid. Figure 3 shows the experimental results using a cantilever with a length of 800 μm and 10% porosity.

The resonant frequency decreases from 117.7 kHz at 10% to 116.5 kHz at 21% humidity. Changing the humidity from 21 to 10% increases the resonant frequency by 1120 Hz and quality factor of the AAO cantilever increases from 61 to 82, respectively (Figure 3).

Using Equation 1 and the Young’s modulus of AAO of 340 GPa one can evaluate the quantity of water adsorbed onto the anodic alumina surface. The calculation gives a result of Δ*m* = 20 pg at a sensitivity Δ*f*/Δ*m* of 56 Hz/pg. On the other hand, the amount of absorbed water can be estimated from the Langmuir monolayer absorption isotherm. Taking into account the total area of the cantilever of 1.74·10^−6^ m^2^ (1.58·10^−6^ m^2^ interior and 1.63·10^−7^ m^2^ exterior area) and the density of the active sites of 4.8 nm^−2^ the maximal mass of a water monolayer adsorbed onto cantilever surface is 250 pg. This results in an increase of the experimental water surface coverage of about 8% within the experimental humidity levels.

From the Langmuir sorption isotherm a humidity change from 10 to 22% should result in growth of the surface coverage by 8.9%, which fits well to the obtained value. However, despite the good agreement between the estimations we should stress that they can be far from being realistic due to a strong chemical interaction of water molecules with AAO surface and pore curvature.

**Figure 4:**
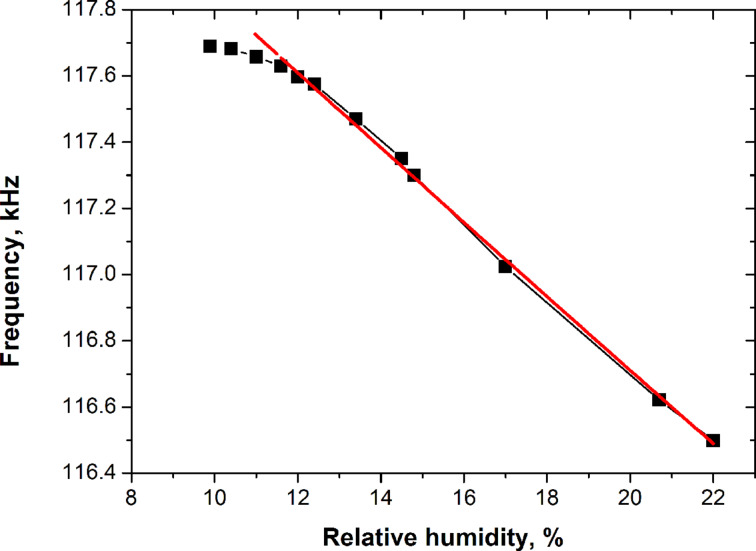
Resonance frequency of the AAO cantilevers (2 μm thick, 800 μm long and 100 μm wide) as function of relative humidity from 10 to 22%. Red line: approximation of the linear part as *f* = 118.9972·(1 + 0.00198*H*)^−1/2^. Inaccuracy of frequency detection is below the size of the symbols.

Figure 4 illustrates the resonant frequency dependence on air humidity. The experimental points were obtained by measuring the resonant frequency with decreasing humidity levels from 22 to 10% (at 298 K).

After each humidity decrement the system was left to reach equilibrium for 10–30 min. Equilibration criterion was resonant frequency creep below 1 Hz/min. The curve indicates a linear behavior approximated by *f* = 118.99·(1 + 0.00198*H*)^−1/2^ (red line), which correlates well with Equation 1. The humidity sensitivity of the AAO cantilever in the linear range estimated by the slope of the experimental curve equals about 100 Hz/%. Hysteresis was not observed for the considered range close to equilibrium. The resonance frequency for the same value of humidity measured after a cycle of increasing and decreasing humidity is well reproduced within the error of 30 Hz. We believe this error corresponds to an inaccuracy of setting up the desired humidity value and a slight deviation from equilibrium during measurements. The plateau at low humidity levels demonstrates a moisture detection limit of 10%. This likely occurs due to residual native water absorbed on the AAO surface, which cannot be removed without heating. Linear behavior of environmental humidity on the resonance frequency of the cantilever oscillation allows us to use AAO cantilevers as humidity sensors at least in the humidity range of 10–22%.

## Conclusion

The proposed combination of anodic oxidation and photolithography processes enables the successful formation of porous alumina cantilevers with desired geometric characteristics. Because of the high surface area of the pores the cantilevers exhibit an exceptional sensitivity to absorbed mass. The resonance frequency shift in water vapor absorption experiments over a humidity range of 10–22% fits well to the monolayer adsorption isotherm. A strong response to environmental change enables the use of AAO cantilever arrays in microsensors with ultra-low detection limits. The study has to be considered as an initial experiment to focus on the profound understanding of micromechanical behavior of anisotropic porous materials having a high specific surface area, such as anodic alumina films.

## Experimental

### Preparation of the cantilever array

**Figure 5:**
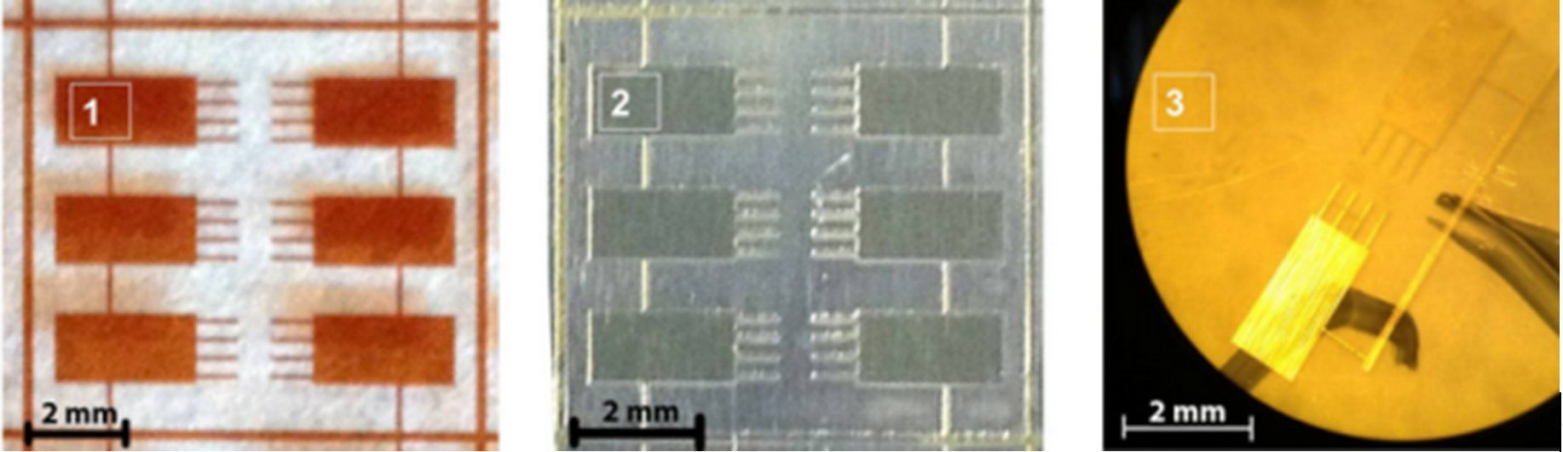
Photomask (1); cantilever arrays as formed on Al-foil (2); free-standing anodic alumina cantilevers (3).

AAO layer formation was carried out in 0.3 M H_2_C_2_O_4_ (98%, Aldrich) at a constant voltage of 40 V. The electrolyte was pumped through the two-electrode cell by a peristaltic pump, and its temperature was kept constant (2 °C) during anodization. The films with a thickness of 2 μm were obtained by controlling the total electric charge. To define the shapes of the cantilever array the pattern of the photolithographic mask was directly transferred onto the AAO surface (spin-coat with S1818 microposit, 6 s at 500 rpm and than 20 s at 5000 rpm, 5 min of drying at 110 °C, 315 mJ of UV radiation [13]). Then unmasked AAO was etched away with 0.5% KOH for 10 min. The mask was removed after drying the samples at 160 °C for 10 min with acetone. Finally the remaining aluminium was dissolved in 0.5 M CuCl_2_ acidic solution (5 vol % HCl). The size of the obtained microcantilevers equals 800 × 100 × 2 µm^3^ (Figure 5).

One should note that these dimensional characteristics can be simply tuned by choosing lithographical mask with the desired cantilever shapes and varying of anodization charge. Films were characterized by optical (Nikon Eclipse 600pol) and scanning electron (LEO Supra 50VP) microscopes. The density of anodic alumina was determined by measuring sample hydrostatic weight.

### Resonance frequency measurement

**Figure 6:**
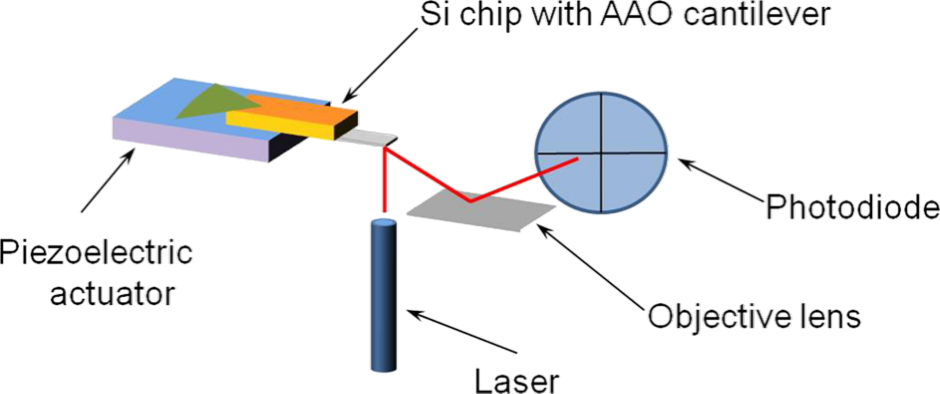
Scheme of measurement cell in atomic force microscope with an integrated optical read-out.

Mechanical measurements with air, vacuum and wet media were carried out by AFM (NTEGRA Aura) in the range of 0–600 kHz. The experimental setup used in this study is shown on Figure 6. The AFM is equipped with vacuum chamber Ar-flow controllers as well as humidity and temperature sensors.

**Figure 7:**
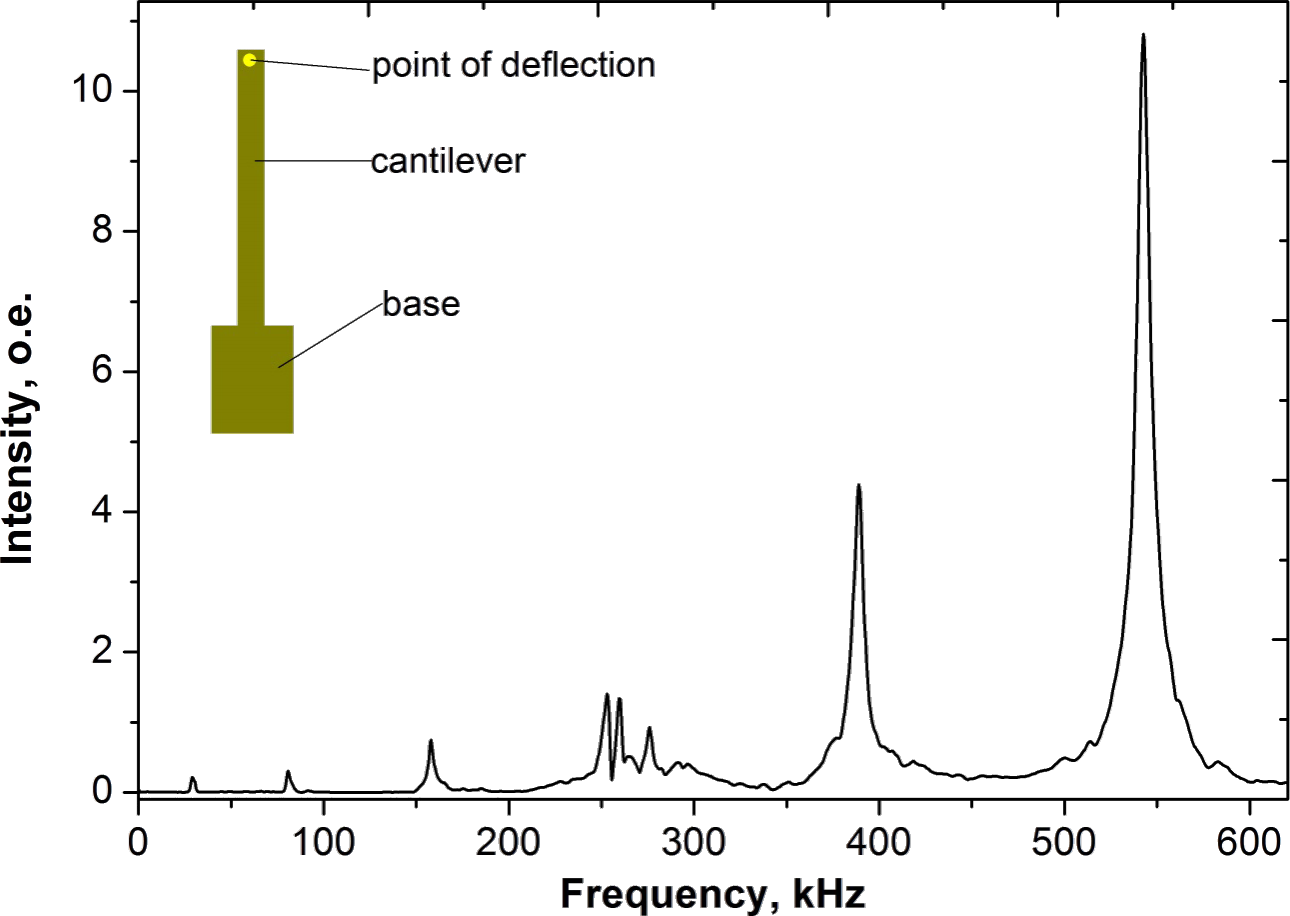
Spectrum of lateral (in-plane) modes corresponding to an alumina cantilever 2 μm thick, 800 μm long and 100 μm wide.

The desired moisture level was obtained by controlling the humidity at 25 °C with saturated salt-water and CaCl_2_ dryer. Saturated salt-water was used to reach high relative humidity in the microscope chamber. After stabilization of humidity equilibrium in the system we start measurements of the resonance frequency over a CaCl_2_ dryer down to the lowest possible humidity level. The microscope determined the upper limit of humidity level. Typically around 10 min have been needed to stabilize the cantilever to a fixed baseline. Humidity levels were varied in the range of 10–22%. An example of the resonance spectrum of porous alumina cantilevers detected with the AFM is shown in Figure 7.

The arrays were supported by Si tips before measurements. The reflectivity of the surface of anodic aluminium oxide is sufficient for detecting vibrations without additional metal reflective coating. According to the exhibited behavior, adequate level of sample reproducibility has been demonstrated by the fabrication procedure described. The frequency does not shift for different points along the one cantilever. In contrast, intensity and a uniform vibration were indicated along the whole cantilever independently from where the AFM beam was detected. The magnitude of the maximal resonance frequency (about 545 kHz) predictably decreases when the optical beam of AFM moves to base of array. Experimentally we established the excitation of mechanical vibrations perpendicular to the cantilever surface as torsion. The thickness of cantilever arrays influences on stiffness and may also cause an increasing in resonance frequency: from 500 kHz for 2 μm thick cantilevers to 670 kHz for 30 μm thick cantilevers.
